# The impact of the COVID-19 pandemic on the attitude towards childbearing of married aged 20–30 Turkish women who are not yet mothers: a cross-sectional study

**DOI:** 10.1186/s12889-023-15976-2

**Published:** 2023-06-01

**Authors:** Cihad Dundar, Tugce Kaya Elverdi

**Affiliations:** Dept. of Public Health, Faculty of Medicine, Ondokuz Mayıs University, Samsun, Turkey

**Keywords:** Childbearing, COVID-19, Fertility, Married, Pandemic, Pregnancy, Turkish

## Abstract

**Background:**

The COVID-19 pandemic has not only changed physical health and the economy, but also changed plans for the future with its impact on social status and mental health. Changes in fertility preferences in many countries are also part of this influence. We aimed to evaluate the effect of the COVID-19 pandemic on attitude toward the childbearing in women.

**Methods:**

This cross-sectional study was conducted on married aged 20–30 Turkish women who are not yet mothers, between January and June 2022, using Google forms. The questionnaire consisted of the sociodemographic data form, The Attitude towards Fertility and Childbearing Scale, and the Fear of COVID-19 Scale. In addition, women were asked about their exposure to COVID-19 and the severity of their illness.

**Results:**

Only one-third of participants said they would like to have children in the next year; 61.2% would consider them later, while 4.5% did not. Common reasons for not intending to have children were “it is early to become a mother (34%)”, “economic difficulties (25.3%)”, and “career plans (16%)”. The fear of COVID-19 scale score, with an arithmetic mean of 16.8 ± 5.5, was found to be significantly lower in women who did not want to have children in the next year than in women who wanted to have children (*p* = 0.042). Except for the profession, there was no significant difference between the COVID-19 fear scores by the participants’ sociodemographic characteristics.

**Conclusions:**

Among the married aged 20–30 Turkish women who are not yet mothers, the rate of those who intend to childbearing was found still low. The main reason for women who did not want to have children in the next year was that they thought it was too early to have a child. Besides low fear of COVID-19 scale scores; economic concerns and career plans, which came in second and third place in the reason list, showed that the fear of infected with SARS-CoV-2 during the pandemic did not affect the women’s attitudes toward fertility.

## Introduction

The measures taken within the scope of the COVID-19 pandemic have caused significant changes globally, affecting not only large systems such as healthcare and education but also individuals and families [1]. The feeling of uncertainty about the future created by the pandemic and the fear of being infected have led to physiological effects in addition to psychological ones [2, 3]. According to the WHO report, based on data from 105 countries, the most frequently disrupted services during the pandemic were facility-based services (61%), non-communicable disease diagnosis and treatment (69%), family planning, and birth control (68%) [4]. A 2020 study by the Guttmacher Institute has shown that the pandemic has affected women’s plans to have children, with 40% of women changing their plans, 41% of women with children worrying about not being able to care for their children, and 33% of women experiencing delays or cancellations of reproductive health services [5]. At the onset of the COVID-19 pandemic, the American Society for Reproductive Medicine (ASRM) and the European Society of Human Reproductive and Embryology (ESHRE) independently recommended that reproductive care be suspended except for the most urgent cases [6]. Thus, the COVID-19 pandemic has created numerous challenges in the area of reproductive health, including family planning and the desire to conceive and have children. The literature has extensively discussed how COVID-19 affects women’s sexual and reproductive health worldwide, but there is limited information available on the situation in Türkiye.

Although coronavirus can infect people of all ages, pregnant women, elderly people and those with chronic illnesses are at higher risk of transmission and mortality [7]. The data on the effects of COVID-19 infection during pregnancy is still limited, and there is no evidence that pregnant women have a higher risk of contracting COVID-19 than the general population [8, 9]. However, it has been reported that pregnant women with COVID-19 are at higher risk for complications such as preterm birth, premature rupture of membranes, preeclampsia, and cesarean delivery due to fetal distress [7, 10]. This may lead to a delay in the desire to have children due to fear of contracting the disease and concerns about pregnancy care [11]. It may be too early to determine its impact on birth rates, but it is expected that the COVID-19 pandemic will cause a postponement in fertility, which can significantly contribute to the increasing uncertainty among people [12]. Therefore, examining women’s attitudes towards childbearing can inform us about how the pandemic and its consequences affect their fertility behavior. Based on the gap in the literature regarding the attitude of Turkish women towards childbearing during the pandemic, our aim was to evaluate the intentions of married Turkish women aged 20–30, who are not yet mothers towards having a child, and their fear of COVID-19. We hypothesized that these women would be delaying their plans or attempts to have children and that those experiencing pandemic-related stress and economic hardship would be more likely to postpone or abandon their pregnancy intentions.

## Materials and methods

### Study design

This cross-sectional survey was conducted on married Turkish women aged 20–30 recruited using various social media platforms such as WhatsApp, Facebook, and Twitter in the period January-June 2022. The questionnaire along with the research purpose was shared with the participants through a Google form.

### Sampling and participants

Inclusion criteria were 20–30 years old, married, and Turkish women who had not yet become mothers. Women who met the criteria and voluntarily agreed to participate in the study were asked to respond to the questionnaire. A snowball sampling method was used to increase the sample size, whereby eligible participants were asked to share the survey link with their female friends with similar qualifications. The minimum sample size was determined to be 245 using the G*Power 3.1.9.2 package program (t = 1.96; d = 0.05, *p* = 0.80). The participants were presented with an online informed consent document, which required them to click on an acceptance box at the bottom of the agreement to indicate their consent to participate before starting the survey. Those who did not provide informed consent were excluded from the survey. At the end of each day, the completed forms were checked for participant eligibility, and the data were transferred to the research database. Women who were single, younger than 20or older than 30, had a health problem preventing conception, or were living outside of Türkiye, were excluded from the study. After reaching the number of 245 eligible participants, we closed the survey links to stop further data collection.

### Measures

#### Demographics

In the first part of the three-part questionnaire, the participants were asked to provide their age, duration of marriage, the highest level of education attained, perceived economic status, profession and employment status, whether they planned to have a child in the next year, and the reasons why they did not want to.

#### Attitudes towards fertility and childbearing

The second part of the survey included the Attitudes towards Fertility and Childbearing Scale (AFCS) created by Söderberg [13]. The Turkish version of the scale was made by Aşçı et al. [14]. AFCS is rated in the five-point Likert type (1 = Totally Disagree and 5 = Totally Agree) and does not contain reverse-scored items. This scale consists of 27 questions, and has three sub-dimensions: “the importance of fertility for the future” (nine questions), “childbearing as a barrier at the moment” (twelve questions), and “female identity” (six questions). Cronbach’s alpha internal consistency coefficients of the sub-dimensions were 0.91, 0.90, and 0.60, respectively. The first sub-dimension, “the importance of fertility for the future”, contains statements about the present and future importance of fertility and motherhood. It captures the idea that both fertility and motherhood are considered indispensable parts of femininity and that having children is an expected stage of life. High scores from this subscale indicate that the respondent wants to become pregnant and is looking forward to having children. High scores from the 2nd sub-dimension, “the childbearing as a hindrance at present” where children were considered to limit women’s personal lives and carry responsibilities that do not fit the current life situation, indicate that the respondent does not currently want to become pregnant and sees having children as a barrier to their economic or career plans. Finally, high scores obtained from the “female identity” sub-dimension indicate that the respondent’s fertility is important for a sense of femininity and unity with other women [15].

#### Exposure and fear of COVID-19

The third part of the survey consisted of the seven-question short form of the fear of the COVID-19 Scale. Each question was scored on a scale of one to five, with a total score ranging from seven to 35. A higher score indicates a greater fear of COVID-19. The scale was originally developed by Ahorsu et al. and was adapted into Turkish by Satici et al. [2]. In the Turkish adaptation study, the internal consistency of the scale and the test-retest reliability were found to be acceptable (α = 0.82 and ICC = 0.72). Participants were also asked whether they had contracted COVID-19 before, the severity of the disease, and whether they had been hospitalized.

### Statistical analysis

We used SPSS 22.0 package program for the statistical analysis of the data obtained from the questionnaires. All parametric continuous variables were presented as arithmetic mean ± standard deviation, and the non-parametric data were presented as numbers and percentages. While Student’s t-test and ANOVA tests were used for comparing continuous variables between groups, Chi square (X^2^) test was used for comparing categorical variables. The Spearman correlation test was used to evaluate the co-variation of continuous variables. The statistical significance level was set at *p* < 0.05.

## Results

The majority of the 245 married Turkish women aged 20–30 who had not yet become mothers were between the ages of 26 and 30. The mean age was 27.6 ± 2.2 years, and 98% of them were highly educated (Table 1). The median duration of marriage for the women was 2 (IQR: 2–3) years. They participated in the study from 46 different cities in Türkiye, mainly metropolitan areas.

**Table 1:** The sociodemographic characteristics of the participants

Characteristics		n		%
Age (Year)				
20- ≤25		25		10.2
> 25–30		220		89.8
Education				
High school		5		2.0
University		240		98.0
Perceived economic state				
Low		12		4.9
Average		180		73.5
High		53		21.6
Duration of marriage				
< 2 years		145		59.2
≥ 2 years		100		40.8
Job/Employment status				
Unemployed		40		16.3
Health care worker		144		58.8
Non-healthcare worker		61		24.9
**Total**		**245**		**100.0**

Of the participants, 84 (34.3%) said they would like to have children within the next year, 150 (61.2%) said they would consider having children later (Table 2).

**Table 2:** The relationship between the desire to become a mother and demographic variables and exposure to COVID-19

		Having a desire to become a mother											
Characteristics		Yes		%		No		%			X^2^		p
Age (Year)													
20- ≤25		19		28.4		48		71.6			0.79		0.495
> 25–30		65		36.5		113		63.5					
Education													
High school		2		40.0		3		60.0			0.78		0.559
University		82		34.2		158		65.8					
Perceived economic state													
Low		2		16.7		10		83.3			2.34		0.311
Average		61		33.9		119		66.1					
High		21		39.6		32		60.4					
Duration of marriage													
< 2 years		42		29.0		103		71.0			4.46		**0.040**
≥ 2 years		42		42.0		58		58.0					
Job/Employment status													
Unemployed		14		35.0		26		65.0			0.08		0.960
Health care worker		50		34.7		94		65.3					
Non-healthcare worker		20		32.8		161		65.7					
Exposure to COVID-19													
Yes		39		40.2		58		59.8			2.49		0.131
No		45		30.4		103		69.6					
**Total**		**84**		**34.3**		**161**		**61.7**					

The rate of those who wanted to have a child in the next year was higher among those who were over 25 years old, had completed high school education, perceived themselves to have s high income, were married for over 2 years, were unemployed, and had been exposed to COVID-19. Out of all the participants, only 11 (4.5%) reported that they did not want to have children at all., The reasons given by 150 women who did not wish to have children in the next year are presented in Table 3.

**Table 3:** Reasons for not wanting children in the next year

Reason		n		%
Thinking it’s early		51		34.0
Economic distress		38		25.3
Career plan		24		16.0
Not feeling ready and competent		12		8.0
Future anxiety		12		8.0
COVID-19 fear		7		4.7
Other		6		4.0
**Total**		**150**		**100.0**

The mean scores of the AFCS subscales were found as 29.4 ± 9.1 for the “importance of fertility for the future”, 42.8 ± 11.8 for the “childbearing as a disability at present”, and 20.5 ± 4.6 for the “female identity”. The distribution of subscale scores according to the sociodemographic characteristics of the participants is presented in Table 4.

**Table 4:** Distribution of AFCS sub-dimension scores according to sociodemographic characteristics (Mean ± SD)

Characteristics		n		Importance of fertility for the future		Childbearing as a hindrance at present		Female identity
Age (Year)								
20- ≤25		25		30.5 ± 9.2		40.4 ± 12.7		19.7 ± 5.4
> 25–30		220		29.3 ± 9.1		43.0 ± 11.7		20.6 ± 4.5
*p*				0.647		0.989		0.824
Duration of marriage								
< 2 years		145		29.1 ± 9.3		42.4 ± 11.9		20.4 ± 5.0
≥ 2 years		100		29.9 ± 8.9		43.2 ± 11.7		20.7 ± 3.9
*p*				0.664		0.529		0.492
Education								
High school		5		30.0 ± 5.8		38.6 ± 13.2		21.2 ± 4.9
University		240		29.4 ± 9.2		42.9 ± 11.8		20.5 ± 4.6
*p*				0.824		0.513		0.775
Perceived economic state								
Low		12		27.5 ± 9.6		46.3 ± 11.7		21.8 ± 5.8
Average		180		28.8 ± 9.2		43.2 ± 11.9		20.4 ± 4.7
High		53		31.7 ± 7.8		40.4 ± 11.6		20.9 ± 3.9
*p*				0.101		0.173		0.486
Job/Employment status								
Unemployed		40		25.4 ± 10.3		40.6 ± 14.8		19.4 ± 6.3
Health worker		144		30.2 ± 8.9		42.1 ± 10.8		20.6 ± 4.3
Non-health worker		61		30.3 ± 8.1		45.6 ± 11.7		21.2 ± 3.9
*p*				** 0.012**		0.072		0.166

The importance of fertility for future scores was found higher in both health care and non-healthcare workers compared to unemployed women (*p* = 0.009; *p* = 0.076, respectively).

Ninety-seven women (39.6%) stated that they had COVID-19. Of the women diagnosed with COVID-19, 13 (5.3%) reported severe disease, 46 (18.8%) mild, and 38 (15%) moderately severe. Only 4 (1.6%) women had been hospitalized due to COVID-19.

The mean score of the fear of COVID-19 scale was 16.8 ± 5.5. There was no significant difference between the scores of the fear of COVID-19 scale regarding participants’ sociodemographic characteristics, except for the profession. The group with the highest fear of COVID-19 scale score was the non-health worker group, while the healthcare worker group had the lowest score (*p* < 0.001). The mean scores of the fear COVID-19 scale, importance of fertility for the future, and childbearing as a disability at present were significantly lower among women who did not want to have children in the next year compared to women who wanted to have children (Table 5).

**Table 5:** Distribution of the Fear of COVID-19 scale and the AFCS sub-dimension scores according to willingness for becoming a mother (Mean ± SD)

	Having a desire to become a mother							
Scale		Yes (n = 84)		No (n = 161)		t		p
The Fear of COVID-19		17.9 ± 5.8		16.3 ± 5.4		2.05		0.042
Importance of fertility for the future		33.8 ± 7.2		27.1 ± 9.2		6.21		< 0.001
Childbearing as a hindrance at present		36.2 ± 8.9		46.2 ± 11.8		7.40		< 0.001
Female identity		21.5 ± 4.4		20.1 ± 4.6		2.37		0.019

We found a positive correlation between “the fear of COVID-19 scale” scores and “the importance of fertility for the future” subscale scores (r = 0.20; *p* = 0.001) and “female identity” (r = 0.16; *p* = 0.013) subscale scores. However, there was a negative-weak correlation between “the fear of COVID-19 scale” scores and “the childbearing as a hindrance at present” subscale scores (r=-0.13; *p* = 0.037).

## Discussion

Worldwide, fertility rates have significantly decreased over the past fifty years, and the maternal age at first birth has risen [16]. According to data from the Turkish Statistical Institute, the total fertility rate in Türkiye decreased from 2.38 to 2001, to 2.05 in 2010, to 1.88 in 2019, to 1.76 in 2020, and to 1.70 in 2021, indicating a permanent decline below the population regeneration level of 2.10 since 2016 [17]. Numerous articles have been published on whether the COVID-19 pandemic has affected this decline, which has also been seen in many high-income countries [18–21].

The COVID-19 pandemic has caused global fear, anxiety, and distress due to its high contagiousness and deadly consequences. Moreover, studies have demonstrated that the mean anxiety and depressive symptom scores are higher than previously reported, and women with high levels of anxiety and depressive symptoms are more likely to postpone their fertility plans [18, 19]. We found that the mean score of the fear of COVID-19 was 16.8. According to the literature, the fear of COVID-19 has been shown to vary greatly across different times and setting. For example in Russia and Belarus, where the same scale was used, it was reported that the fear of COVID-19 score was 17.2, and university students had higher scores than those who graduated, and women had higher scores than men [22]. The fear of COVID-19 scale score varies according to sociodemographic factors such as religion, country of residence, gender, and academic status. For example, in Spain, the score was determined to be 16.8, in Iran it was 27.4, and in Saudi Arabia, it was 17.0 [22–24]. In our study, the mean score of the fear of COVID-19 scale was 16.8, which is relatively lower than that reported in other studies. This may be attributed to the fact that the majority of our participants were highly educated and healthcare workers. Moreover, during the study period in 2022, the increasing number of people vaccinated in Türkiye and worldwide, the decreasing incidence of COVID-19, the measures taken against COVID-19, the nature of the information shared with the community about the infectious disease, and the relaxation of pandemic measures may have also contributed to the reduction of individual anxiety.

Contrary to our findings studies conducted in different countries such as China, India, and Mexico during the peak of the pandemic showed that health care workers had higher average scores on the fear of COVID-19 scale than non-health care workers, which supports this hypothesis [25, 26].

The main reasons for the decline in fertility rates, principally in high-income countries, are the educational status of women, their increasingly active role in the labor market, and an incomplete “gender revolution” [27]. The double burden of working life and housework causes women to limit their fertility [28]. As revealed in the COVID-19 pandemic, the adverse effects of uncertainty in the economy on fertility have been shown by numerous studies in all income groups among various countries [21, 29]. The fact that one in four women in our study stated that they did not want to have children in the next year directly because of economic concerns seems to confirm these hypotheses. We also found that women who perceived their economic situation as medium or poor had lower scores on the “importance of fertility for the future” subscale than those who were in good financial condition.

From a different perspective, it can be argued that the increase in economic uncertainty in high-income countries will reduce fertility. In low-income countries, reduced access to family planning and social restrictions that required people to spend more time at home may lead to an increase fertility. However, the pandemic has had positive impact, albeit not significant, on the crude birth rates of nine countries [30]. These are Slovenia, South Korea, Norway, Denmark, Finland, Sweden, Germany, the Netherlands, and Switzerland. On the other hand, it is crucial to assess women’s fertility preferences without confounding factors such as the country’s economic situation, attitudes, and their mental and physical health status. Therefore, it should not be expected that the fertility consequences of the pandemic would be homogeneously distributed domestically or globally. For example, in Europe, fertility intentions have been shown to decline differently in each context. In Italy, fertility intentions declined among people under 30 and highly educated, while in Germany, it was among those living in areas with the highest infection rates [20, 29]. Particularly in Southern Europe, where youth and women’s employment and fertility levels are the lowest, the uncertainty caused by the COVID-19 pandemic may have been exacerbated by the ongoing impact of the 2008 financial crisis. Only in Germany, France, and the United Kingdom has it been reported that those living in regions with a high cumulative prevalence of COVID-19 cases have changed their fertility plans. In ‘red’ regions, where the spread of COVID-19 is high, the proportion of those who are still planning to have children is lower than in other regions. On the other hand, the proportion of those who have stopped planning in Germany and those who have postponed planning in the United Kingdom was found to be higher [29].

Our study has some limitations. Firstly, our results cannot be generalized to all Turkish women, as we could not obtain a sample that representative of the society using the snowball sampling method. Secondly, the cross-sectional design we used cannot establish causality regarding the findings. Lastly, not having knowledge about the vaccination status is another limitation.

## Conclusions

Our results showed that two-thirds of married Turkish women aged 20–30, who are not yet mothers, did not intend to conceive in the next year. However, very few women attributed this to the fear of COVID-19. It was observed that most women’s attitudes towards fertility were influenced by economic concerns and career plans. Relatively high scores on the “Importance of fertility for the future” and “Female identity” subscales indicated that the pandemic might lead to a temporary decrease in the number of women actively trying to conceive, instead of a permanent decline in fertility rate of Turkish women. Longitudinal and qualitative studies are necessary to gain further insight into changes in both fertility behavior and the underlying dynamics affected by the pandemic.

## Data Availability

The datasets used and analyzed during the current study are available from the corresponding author on reasonable request.

## Abbreviations

AFCS: Attitudes towards Fertility and Childbearing Scale
COVID-19: The coronavirus disease 2019
